# How to Prevent the Spread of Contagious Diseases

**Published:** 1908-01

**Authors:** Samuel A. Visanska

**Affiliations:** Atlanta, Georgia; Visiting Physician to Home for Incurables, and Physician-in-charge of Children’s Dispensary of the Woman’s Board of City Missions


### Journal-Record of Medicine

Successor to Atlanta Medical and Surgical Journal, Established 1855, and Southern Medical Record, Established 1870.

OWNED BY THE ATLANTA MEDICAL JOURNAL CO.

**Published Monthly.**

*Official Organ Fulton County Medical Society, State Examining Board, Presbyterian Hospital, Etc.*

VOL. IX. JANUARY, 1908. No. 10

BERNARD WOLFF, M. D., EDITOR, 319-320 Prudential Bldg.

GEORGE BROWN, M. D., BUSINESS MANAGER, Business Office 1½-5½ S. Broad St.

E. G. BALLENGER, M. D., ASSOCIATE EDITOR AND ASSISTANT BUSINESS MANAGER.

### ORIGINAL COMMUNICATIONS.

### HOW TO PREVENT THE SPREAD OF CONTAGIOUS DISEASES.

By Samuel A. Visanska, Ph. G., M. D., Atlanta, Georgia.

Visiting Physician to Home for Incurables, and Physician-in-charge of Children's Dispensary of the Woman's Board of City Missions.

If every professional man in the land were asked to unite in framing some one general rule by which to further the public good, I believe that all would agree that "Co-operation and Protection" would best cover the question. Of course, for each profession this rule would have a different significance, but from the standpoint of the physician, it must mean "protection" of the public health, just as to the lawyer it must mean protection of public property, or to the minister, protection of the public morals.

But to the physician, it is almost a sacred duty to co-operate with the City Board of Health in a unanimous effort to protect the public from contagion, and to insure, wherever possible, public sanitation and immunity from the spread of contagious disease. We, of Atlanta, are fortunate in having a most efficient Board of Health to guard the public lives, but no set of laws, however wise, can reach all cases nor all classes, and no rule, however rigid, can accomplish desired ends, without the fullest co-operation of the people for whose benefit they are designed. Each department or division of our city life must face a different set of health problems, but I know of no class to whom the problem is presented in a more complex form than to the teachers and directors of our public school system in general and to the heads of Kindergarten Departments in particular. First of all, the mere fact of a woman's having selected Kindergarten work as her vocation, argues that she has within her heart of hearts, a deep love for little children—a love which proves her kinship with Nature, the great Mother, and which is an emotion that provides for the protection, as well as for the perpetuation, of the race. While we must admit that every adult owes a certain duty toward the little children of the world, yet no class is performing this duty more grandly or more wisely than is the teacher in the Kindergarten. To them all help is due in their great and noble undertaking, and I am glad of an opportunity to aid them as far as I am able to guard the well children under their care from the manifold dangers of contagion, which, in the very nature of things they are daily and hourly subjected. In this work, the public, the teachers and the physicians of Atlanta are materially aided by having the wise assistance of the present Board of Health—an organization which leaves nothing undone to promote public welfare.

But it is first necessary to understand and to appreciate the great need of this vast work of public protection, and then for each citizen, teacher and physician in the community to unite in a concerted effort to avert contagion, wherever possible, by keeping sick children apart from well ones; by watching ailing children for fear they may become sick, and by immediately isolating every suspicious case of any kind until it shall develop, either into a real illness of more or less seriousness, or shall prove to be merely a passing indisposition.

Strange to say, however, the greatest obstacle against the proper protection of the public is the public itself! To the ignorant or the uninformed, this may be readily forgiven, but we all must know of cases where even the enlightened and intelligent members of a community actually resent the intervention of the Board of Health, and even censure the physician who dares to declare that there exists a case of contagious illness in the family, and who, having discovered such a case, insists upon reporting it to the city Board of Health. The only explanation of this attitude is that these people are suffering from an exaggerated form of selfishness. They do not consider the danger to others from the innumerable sources of infection; they consider only the personal inconvenience of having their well children kept from school because one of their number happens to be ill; and the petty, personal discomfort of being themselves isolated from social intercourse outweighs with them the grave dangers of the life or death of their associates. This attitude cannot be too highly censured, yet it is one with which every physician meets and with which his best efforts, or even the stringent laws of his city, cannot successfully cope, nor altogether overcome.

Parents are positively forbidden to send children to the public schools from a home in which there exists a contagious disease; but what can be done if this disease is carefully concealed?

And again, in cases where the contagion is known and the children are kept from the public schools, how many are also kept from private schools, from Sunday schools, or from public places, such as theatres, parks and stores? Lack of consideration for others in cases such as these is the chief cause of the spread of contagion.

Yet in considering the spread of contagious diseases it is of paramount importance to remember that many persons are uninformed both as to the many possible sources of danger, as well as being actually ignorant as to the necessary precautions to be taken after the disease is present, and also as to the methods of preventing certain kinds of diseases. To the teachers of young people is given a rare opportunity to explain many of these conditions and to impress on plastic young minds many facts which will be of interest and importance and which, through the medium of the children, are carried into the homes and minds of parents or guardians.

Of all the interesting studies of contagious diseases, there is no one which presents more food for thought than the history of small pox, and the great preventative, vaccination. Even at this date, there are many persons who disapprove of vaccination, and one can only believe that this attitude is the result of ignorance of the great boon given to humanity by Edward Jenner, the discoverer of vaccination. When this young man was pursuing his professional studies at Sodbury, a young country woman, on hearing small pox mentioned ,declared within the hearing of the young student, "I cannot take that disease for I have had cow pox." This incident made a deep impression on the mind of Edward Jenner, and may be said to have been the awakening impulse which, after years of patient study and experiment, culminated in a discovery which has conferred one of the greatest known benefits upon the human race.

To properly appreciate the life-saving value of Jenner's discovery, it is necessary to know something of the fearful mortality of small pox in the pre-vaccination days. It was the most dreadful of all scourges, not excluding the plague, for that disease came but rarely, while small pox was always present. In 1802, a committee of the House of Commons was appointed to investigate the petition of Jenner for a Parliamentary Grant, and, in an eloquent speech on the subject, Admiral Bentley, Chairman of this Committee, said: "The discovery is unquestionably the greatest discovery ever made for the preservation of the human species. It has been proven that in the United Kingdom alone 45,000 persons die annually of the small pox, and throughout the world what is it? Not a second is struck by the hand of time but a victim is sacrificed on the alter of this most horrible of all diseases—small pox."

King Frederick William III of Prussia stated in 1803 that 40,000 persons succumbed annually to the small pox in his Kingdom.

The French Minister of the Interior in reporting on vaccination in 1811, estimated that 150,000 persons died annually in France of the same disease. In Russia, small pox is authoritatively declared to have destroyed 200,000 lives in a single year. I might continue to give even more startling statistics of the enormous death rate from this terrible ill, while disabilities from the disease are almost equally numerous. The early records of the London Asylum for the indigent blind show that three fourths of the inmates had lost their sight from small pox.

From such statements as the foregoing, it will be easy to understand how a physician and a scientist was eager to grasp at any possible alleviation for such a dreadfull ill.

The fact that cow pox secured protection against small pox appears to have been noticed as early as the middle of the 18th. century, by dairy men in England who observed that when small pox prevailed those who had been accidentally infected with matter exuding from certain sores known as "cow pox" which often appeared on the teats and udders of cows, resisted the infection of small pox. It is said that Benjamin Jesty, a Westminster farmer, was the first person in England to employ cow virus against small pox. In 1774, he vaccinated his wife and children with matter taken from the teats of cows that had cow pox. In a week the sores became inflamed and some constitutional disturbance was present. The children were later inocculated with small pox without result. It was cases such as these which led Dr. Jenner to apply the principles of vaccination in scientific form with the results already known. From 1801 to 1822, he received not less than 28 diplomas from institutions of learning and scientific societies in every country of Europe and in the United States and Canada. Can we longer question the marvelous protection afforded by vaccination after the hearty endorsement of the entire scientific world?

There are, however, certain precautions that should always be used in vaccination. The human virus should never be used but once; each vaccination being done from the fresh bovine or calf lymph. In order that a vaccination may pursue a perfect course free from subsequent complications, it is important that certain sanitary precautions be observed. These may be classed as follows;—1. Purity of vaccine virus; 2. Condition of the vaccinee; 3. Asepsis during the insertion of the virus, and subsequent protection of the vaccine lesions; 4. Bovine purity, or a healthy condition of the animal from which the lymph is taken.

If a child be exposed to the liability of contact with small pox, there should be no hesitation in the performance of vaccination, no matter what the condition of the child may be. Scores of children have been vaccinated even while suffering from scarlet fever and diptheria in the hospitals, in order that small pox might be averted, and the results have not been in the least injurious.

The ages at which children should be vaccinated differ in the opinion of physicians, and also in different countries. In Germany, the law requires that every child be vaccinated during the first year, and before possible exposure to infection. Between four and six years, however, is a suitable time, although there have been cases vaccinated at birth. It is best, however, to delay the vaccination of a poorly developed or badly nourished child, especially if it has the slightest tendency to glandular or scrofulous troubles.

Perhaps the most contagious disease to be considered after small pox is scarlet fever. This ill is conveyed in two ways; first, directly from scarlitina patients to the newly infected subject, and second through the intermediation of infected objects. The vast majority of cases doubtless result from exposure to persons suffering from the disease, either in its incipiency, or during the latter part of the disease when the skin is scaling or "peeling off."

The sources of infection from this ill are numerous and in many instances, obscure. I know of a case where a child contracted scarlet fever in a city where there were but a few other cases known to exist, the source of the infection being traced to a carriage in which the child had ridden and which was afterwards discovered to have been used in a funeral procession, the dead child having had scarlet fever. It was supposed that a nurse or relative must have occupied the same carriage in which the well child afterwards rode.

From this it will be seen that infection clings to such articles as bedding, clothing, books, letters, toys, etc. Of the latter, the popular "Teddy Bear" is a fruitful source of contagion, it's long hairy covering being an admirable resting place for germs, as is also the covering of "wooly" animals, which so many children use and enjoy. The "Teddy Bear," however, is perhaps the worst germ preserver of them all and its use should be discouraged on this account.

Letters are another prolific source of infection and all communications from infected rooms should be destroyed immediately or better still, no written messages should be sent out at all.

The ages at which children are most liable to infection from scarlet fever are from two to five years. Negroes are less susceptible to this disease than whites.

It has been stated that milk is sometimes a source of infection in scarlet fever.

Of course, in this disease the most rigid isolation is necessary and there really should be hospitals provided for the treatment of scarlet fever, as well as for the many other contagious diseases, the ravages of which every one knows, though perhaps no class recognizes the dangers more keenly than do the teachers in our schools; more especially, those in the Kindergartens, who are constantly thrown with children of the age most liable to contract this and kindred ills.

An imperative necessity in the treatment and proper care of all contagious diseases is the hygiene of the sick apartments, which not only hastens the recovery of the patient, but also protects others. As soon as it is recognized that a person is stricken with a contagious disease, he should be immediately isolated from the other members of his family. It is best to select a rear room in the home for the treatment of a contagious ill, but the facilities for ventilation should be carefully considered, as it is of importance that there should be plenty of fresh air in a sick room at all times. This does not mean cold air, but the room may be kept at a certain fixed temperature, say 70 degrees, even if there be at least one window kept open constantly. In preparing a room for the reception of a contagious disease, all carpets, draperies, and ornaments should be removed before occupancy by the patient. The family physician usually directs this, but some physicians are more careful in their directions than others. The door leading into the corridor from the sick room and thus communicating with the remainder of the house, should be covered with a sheet kept moist with an antiseptic solution, and this sheet should only be lifted when it is absolutely necessary for persons to pass in and out. Attendants on a contagious disease should exercise care in the changing of their own clothing before going among persons, and it is best to have an entire change of outfit in some adjacent room, which is, if possible, also isolated. Only one person should care for a patient with a contagious disease, or when it is found necessary to have two attendants, both should be rigidly quarantined, even when not on duty in the sick room.

While it is usually considered that a person once having a contagious disease is "immune" from further attacks, this is not an invariable rule; and I know of a case where a physician lost his only child from a second attack of scarlet fever, because he felt safe in being with the child after he had attended other scarlet fever cases and before he had taken the precaution to change his clothing.

It is always safer not to permit a well child to remain in the room with a sick one, and on no account should a sick person have any one occupying the same bed with him. It is oftener easier to care for a sick child during the night if the mother sleeps beside it, but it is actually wrong in sickness and is even to be deprecated in health. Another contagious disease which may be said to be in the same general class with scarlet fever is diptheria, which is equally contagious and against which the same precautions are necessary. In all cases of sore throat there should be a careful examination and a test made to determine the presence of Klebs-Loeffler bacilli, should the throat seem very bad. This is the germ which produces the deadly ill, diphtheria. Membranous croup is another contagious disease similar to diphtheria, but differing from it in the fact that the membrane which forms is farther down the throat, covering the larynx and often causing suffocation if not detected and removed. This disease is also most contagious and, in fact, the same is true of all diseases which affect the mucous membranes, even a common cold. The evidence of the contagious character of the latter is often noted by teachers who observe that a cold will attack a whole roomful of children. Plenty of pure, fresh air is the best preventative of this, but even with every possible precaution, schools must remain the main causes of epidemics among children. Teachers cannot watch their little charges too carefully in order to protect them from contact with diseased children.

Any constitutional predisposition to disease, or any systemic weakness will, of course, make a child more susceptible to contagious diseases, but often perfectly healthy children succumb more readily to contagion than weaker ones, which only emphasizes the fact that all children should be protected from contact with disease at all times.

In treating of contagious diseases it is impossible not to devote much time and attention to tuberculosis, a disease which attacks both young and old and which has done, and is doing as much to depopulate the world as did small pox in the early days, or the plague some centuries earlier. Only within the comparatively recent past has tuberculosis been recognized as a contagious disease, and even now, not nearly as much precaution is taken as the conditions demand. One of the chief sources of infection from tuberculosis is kissing, and for this reason the indiscriminate kissing of children by strangers should be absolutely prohibited. Again the habit of careless expectoration should be strongly condemned, as it is through the agency of the sputum carelessly deposited in the streets, street cars and other public places, that the germ of tuberculosis is carried broadcast throughout the world.

It is of interest to note that the people are awakening to a sense of responsibility in the prevention of contagion and one admirable improvement in the conducting of the schools today can not be too highly commended; this is the almost universal passing of the slate and the many unclean and unsanitary habits that followed the use of a slate, which only required a moistened finger passed across its surface to make it "clean" again. This method of disseminating disease can not fail to be obvious in its results.

Another precaution which suggests itself strongly to the thinking man who would prevent the spread of disease, is a supervision of the homes of wash women. In the South particularly, family washing is done almost exclusively by negro women at their own homes. It will be seen at once what a fruitful source of disease such a custom can, and possibly does become. There should be a state law regulating this work and requiring that every person doing washing at her own home should register with the Board of Health, and should also be required to have her home subject to inspection by the health officers. Those having clean homes will not object to this, and those whose homes need sanitary cleaning will be compelled to do the necessary work in order to meet the requirements of the law. This precaution need not cost the individual wash women a single cent, and could be arranged for with but little extra expense to the Health Department; while it would certainly be of inestimable value in preventing the spread of disease. There should also be a stringent law covering the taking of clothes from infected homes before they have been soaked in a strong antiseptic solution, rendering them harmless even when washed with other clothes.

While it is obviously impossible even to mention here the long list of contagious diseases, yet the majority of the minor ones are well recognized by every one. Every teacher knows the spread of measles, whooping cough and chicken pox after the first case or two is reported in the schools. While these diseases need not be considered dangerous in character, yet measles, especially, carries with it a long train of ills if not carefully treated, and a little sufferer may find himself with weakened eyes, limbs or lungs after a case of measles which had not been properly cared for and the convalescence of which had not been guarded from cold or exposure.

But when the final word is said as to the preventing of contagious diseases, the doctor can only caution against the manifold dangers from myriad sources and advise as to the protection against them. The real benefit to the individual must come from the intelligent co-operation with the health officers of those having the care of others, and I believe this will be granted in fullest measure to the physician, as well as to the Board of Health, as soon as the necessity for it is fully realized by the very capable young women now before me—the teachers in the Kindergartens of Atlanta.

